# TNAP—a potential cytokine in the cerebral inflammation in spastic cerebral palsy

**DOI:** 10.3389/fnmol.2022.926791

**Published:** 2022-09-14

**Authors:** Xiao-Kun Wang, Chao Gao, He-Quan Zhong, Xiang-Yu Kong, Rui Qiao, Hui-Chun Zhang, Bai-Yun Chen, Yang Gao, Bing Li

**Affiliations:** ^1^Research Center for Clinical Medicine, JinShan Hospital, Fudan University, Shanghai, China; ^2^Department of Rehabilitation, Children’s Hospital Affiliated to Zhengzhou University, Henan Children’s Hospital, Zhengzhou Children’s Hospital, Zhengzhou, China; ^3^Henan Key Laboratory of Children’s Genetics and Metabolic Diseases, Zhengzhou, China; ^4^College of Acupuncture-Massage and Rehabilitation, Yunnan University of Traditional Chinese Medicine, Yunnan, China

**Keywords:** spastic cerebral palsy, hypoxic ischemic encephalopathy, TNAP, ALPL, proteomics

## Abstract

**Objective:** Several studies have shown the significance of neuroinflammation in the pathological progress of cerebral palsy (CP). However, the etiology of CP remains poorly understood. Spastic CP is the most common form of CP, comprising 80% of all cases. Therefore, identifying the specific factors may serve to understand the etiology of spastic CP. Our research aimed to find some relevant factors through protein profiling, screening, and validation to help understand the pathogenesis of cerebral palsy.

**Materials and methods:** In the current study, related clinical parameters were assessed in 18 children with spastic CP along with 20 healthy individuals of the same age. Blood samples of the spastic CP children and controls were analyzed with proteomics profiling to detect differentially expressed proteins. On the other hand, after hypoxic-ischemic encephalopathy (HIE) was induced in the postnatal day 7 rat pups, behavioral tests were performed followed by detection of the differentially expressed markers and inflammatory cytokines in the peripheral blood and cerebral cortex of the CP model rats by Elisa and Western blot. Independent sample *t*-tests, one-way analysis of variance, and the Pearson correlation were used for statistical analysis.

**Results:** Through proteomic analysis, differentially expressed proteins were identified. Among them, tissue-nonspecific alkaline phosphatase (TNAP), the gene expression product of alkaline phosphatase *(ALPL)*, was downregulated in spastic CP. In addition, significantly lower TNAP levels were found in the children with CP and model rats. In contrast, compared with the sham rats, the model rats demonstrated a significant increase in osteopontin and proinflammatory biomarkers in both the plasma and cerebral cortex on the ischemic side whereas serum 25 hydroxyvitamin D and IL-10 were significantly decreased. Moreover, serum TNAP level was positively correlated with serum CRP and IL-10 in model rats.

**Conclusion:** These results suggest that TNAP is the potential molecule playing a specific and critical role in the neuroinflammation in spastic CP, which may provide a promising target for the diagnosis and treatment of spastic CP.

## Introduction

Cerebral palsy (CP) is the most common neurological dysfunction in children, frequently accompanied by cognitive, language, and behavioral defects, and can be secondary to epilepsy and musculoskeletal problems (Chambers et al., 2017). CP may be brought about by brain injuries in the majority of children. The pathological changes in CP may be characterized by liquefaction necrosis and astrogliosis in the cerebral cortex, white matter, basal ganglia, and the cerebellum (Sanches et al., 2021).

Clinically, depending on the type and distribution of motor abnormalities and the location of the brain injuries (Johnson, 2002), CP can be classified as spastic, dyskinetic, or ataxic. Spastic CP is the most common form of CP, comprising 80% of all cases (Vitrikas et al., 2020). At present, the etiology of CP still remains unclear, making its clinical diagnosis difficult (Skoutelis et al., 2020). It has been revealed by several research that spastic CP is closely related to inflammation, cellular energy depletion, excitotoxicity, and oxidative stress which are caused by premature delivery, hypoxia, and intrauterine infections (Novak et al., 2017; Galea et al., 2019). There is also evidence that CP may be associated with the persistence of chronic inflammatory processes in the central nervous system (CNS; Yoshida et al., 2019). Multiple factors involved in chronic inflammation, such as cytokines, angiogenic factors, chemokines, and oxidative stress markers, have been implicated in the pathogenesis of CP. Several molecules, including interleukin (IL)-1β, IL-6, and tumor necrosis factor (TNF), have been observed more frequently in the case of CP (Cordeiro et al., 2016; Magalhaes et al., 2018). In recent years, modern mass spectrometry-based proteomic methodologies have high sensitivity capable of identifying low abundant proteins over wide dynamic ranges, consequently identifying novel disease-specific biomarkers and providing biological annotations for disease stages (Di Falco, 2018). Therefore, we hypothesize that critical neuroinflammatory signals play a specific and critical role and mediate the pathological progression in the neuroinflammation in spastic CP, providing a promising target for diagnosis and treatment of spastic CP.

## Materials and Methods

### Study participants

A total of 18 children with CP aged 8–26 months were enrolled in this study from January 2019 to September 2021. According to the European Cerebral Palsy Monitoring Group’s classification system (Sik et al., 2021), the participants were diagnosed with spastic type CP (ST group; 18 participants). The diagnoses and classifications of the children were determined by two independent clinicians. Meanwhile, 20 children aged 8–26 months, with no confirmed neurodevelopmental disorders after careful physical examinations, were included into the study as healthy controls (Table 1). The subjects involved were free from genetic disorders, brain disorders, and metabolic disorders. The research protocol of this clinical study was approved by the ethics committee of Henan Province Children’s Hospital. Informed consent was obtained from the parents or the legal guardians of the child prior to the onset of the study.

**Table 1:** Clinical characteristics of our cohort.

		ST (*n* = 18)	Control (*n* = 20)
Sex	Male	9	10
	Female	9	10
Age in months		8–26	8–25
Weight, kg		9.03 ± 1.62	11.26 ± 1.31
Enhanced muscle tension		+	−
Decreased muscle tension		−	−
Hyperreflexia		+	−
Stereotypical movements		−	−

### Proteomic analyses

The sample we tested was plasma. Four spastic CP samples and four control samples were used for proteomic analysis. A 100 μg protein sample was taken from each sample and digested overnight at 37°C. Each sample was processed and labeled with the instruction of iTRAQ Reagent-8 Multiplexing Kit (AB Sciex, UK). The labeled sample was mixed in equal volumes, desalted, and lyophilized. High-performance liquid chromatography was performed with a Rigol L3000 system and a C18 chromatographic column (Waters BEH C18, 5 mm). A Diane NCS3500 system (Thermo Science FicTM) equipped with a trap and an analytical column was used to fractionate the labeled samples, and the precursor ions decomposed by the higher-energy C-trap dissociation (HCD) method were sent to a tandem mass spectrometry Q Exactive HF-X MS (Thermo Fisher, Waltham, MA) for data acquisition and analysis.

### Clinical parameters of cerebral palsy participants

#### Gross motor function classification

The Gross Motor Function Classification System for cerebral palsy-Extended and Revised (GMFCS-ER; Piscitelli et al., 2021) was used for gross motor function classification. The subjects involved in this motor function assessment covered two different age groups (0 < 2 and 2–4 years old). The motor indicators required by this system included maintaining erecting head, turning over, sitting alone, crawling, standing, and walking. According to motor function performance, the motor dysfunction was divided into five grades, grade I indicating the slightest gross motor dysfunction, and grade V indicating the worst. In sum, GMFCS grades I to III were classified as mild to moderate motor dysfunction, and GMFCS grades IV to V were classified as severe motor dysfunction.

#### Neuroimaging examination

All brain MRI scans were acquired on a 3.0T MR scanner (Discovery MR750, GE Medical Systems, USA). Imaging sequences included transverse T1-weighted (T1W), T2-weighted (T2W), T2-fluid attenuated inversion-recovery (FLAIR), and sagittal T1W imaging, with a section of 4–5 mm thick. No enhanced scanning was performed in all cases. The obtained brain MRI images were evaluated by the pediatric neuroradiologists and CP specialists participating in our study. The findings were divided into periventricular white matter injury (PWMI): periventricular leukomalacia (PVL), ventriculomegaly; diffuse brain injuries: subcortical softening foci, myelin dysplasia, cerebral atrophy, basal ganglia/thalamic lesions; focal lesions: focal cerebral ischemia, porencephalia; cerebral dysplasia: dysplasia of the corpus callosum, cerebellar dysplasia; and normal brain MRI imaging.

#### Additional blood sample examinations

Three-milliliter venous blood was taken from all subjects on an empty stomach in the morning. An ADVIA2400 automatic biochemical analysis instrument produced by German Siemens was used to assess the percentage of lymphocytes (LYM) in the blood. An i-CHROMA Reader immunofluorescence analyzer from Boditech MED Inc., South Korea, was used to determine the C-reactive protein (CRP) level. A Roche Cobas e602 analyzer (Roche Diagnostics, Switzerland), was used to detect 25 hydroxyvitamin D [25 (OH)D], and creatinine (Cr).

### Animal models

Forty-six 3-day-old Sprague Dawley pup rats were acquired from Shanghai Jihui Experimental Animal Breeding Co. Ltd. The pups were fed by their mothers until weaning. All rats were reared in an animal room with sufficient water and food under a 12-h light-dark cycle. The animal experiments were conducted in accordance with the National Institutes of Health Guide for the Care and Use of Laboratory Animals and the 3R principle.

**Figure 1:**
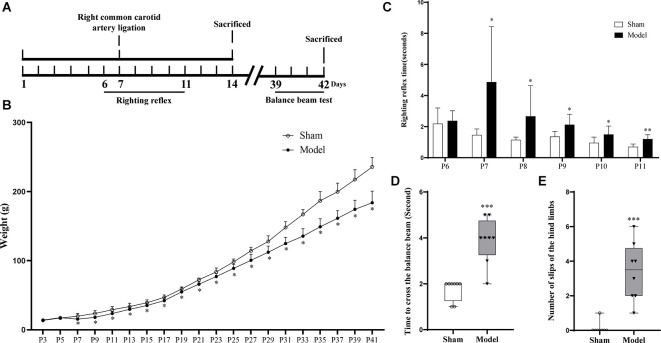
**(A)** Flow chart of the HIE animal model experiments. **(B)** After the operation, the Model group showed continuously significantly less weight than the Sham group (*p* < 0.05, *n* = 10). **(C)** The Model group showed significantly longer righting reflex time from P7 to P11 than the Sham group (*p* < 0.05, *n* = 8). **(D)** Compared with the Sham group, the model group showed a longer time to pass the balance beam and **(E)** higher frequency of leg-slips (*p* < 0.001, *n* = 8). *Denotes statistical significance. *p < 0.05 vs. Sham. **p < 0.01 vs. Sham; ***p < 0.001 vs. Sham.

Forty pups were randomly divided into Sham and Model groups (*n* = 20). On postnatal day 7 (P7), the Model group was subjected to an operation to construct the hypoxic-ischemic encephalopathy (HIE) model, while the operation without occlusion of the artery was performed on the Sham group on the same day (P7); 10 pups from each group were sacrificed on the 7th day post-operationally, and the remaining 10 pups were sacrificed on the 35th day post-operationally. Another eight pups were sacrificed 48 h after the operation for triphenyl tetrazolium chloride (TTC) staining and Nissl staining. The pups of each group were weighed every other day. The righting reflex test was performed from P6 to P11, and the balance beam experiment was performed from P39 to P42 (Figure 1A). Behavioral testing and collection of animal samples were all conducted between 8:00 am and 3:00 pm.

### HIE model construction

The HIE model CP has been widely accepted and recognized as an animal model of CP (Rice et al., 1981). And the operation processes were as follows: on P7, the Model group pups were anesthetized with isoflurane, and the right common carotid artery was ligated. After waking, the pups were placed in a cabin with 8% oxygen and 92% nitrogen for 2.5 h. Finally, the pups were sent back to their mother. As for the Sham group, the common carotid artery was isolated without ligation.

### Behavioral test

Righting reflex test: The pups were placed on their back on a platform and the time for successful righting was recorded. Balance beam test: After three consecutive days of training, the pups successfully passed a crossbar placed 50 cm above the ground, which was 2 cm broad and 120 cm long. On the 4th test day, the time to pass the crossbar and the number of slips of the hind limbs while passing the crossbar were recorded.

### TTC and Nissl staining

**TTC staining**: The sham and model pups were euthanized 48 h post-HIE model creation. After the heart was perfused with normal saline, the pup brains were dissected to make 2 mm thick sections for TTC (Sigma Aldrich, USA) staining.

**Nissl staining**: At 35 days post-HIE model creation, the pups were perfused and fixed after being sacrificed, and the brains were removed for sectioning. After rehydration, the brain slices stained with 0.5% tar violet (Macklin, China), were observed under an OLYMPUS-BX51microscope (OLYMPUS, Japan) and photographed with the cell Sens Standard 1.12 (OLYMPUS, Japan).

### ELISA

Fresh plasma was collected from children and centrifuged at 3,000 rpm, 10 min. On the other hand, after the rats were anesthetized, arterial blood was collected from the abdominal aorta into an EDTA anticoagulation blood collection tube, and plasma was obtained *via* centrifugation at 3,000 rpm/min for 10 min. Human tissue nonspecific alkaline phosphatase (TNAP; ml906210V), rat tissue nonspecific alkaline phosphatase (TNAP; ml497021V), Rat 25 (OH)D (ml038318V), Rat osteopontin (OPN; ml003147V), Rat C-reactive protein (CRP; ml038253V), Rat interleukin (IL)-6 (ml102828V), Rat IL-10 (ml002813V), and Rat IL-17 (ml003003V) were detected according to the Elisa kit (Enzyme-linked Biotechnology Co., Ltd, Shanghai) manufacturer’s recommended procedure.

### Western blot

The expression levels of TNAP, NF-κB, IL-10, and IL-6 in the right cortex of the pups on the 7th and 35th days post-HIE modeling were measured by Western blot. The primary antibodies used were rabbit anti-TNAP (DF6225, Affinity), mouse anti-IL-6 (ab9324, Abcam), rabbit anti-NF-κB (#8242, Cell Signaling Technology), and rabbit anti-IL-10 (ab9969, Abcam). The internal control was rabbit anti-β-Actin (ab8227, Abcam). The gray values were quantified and analyzed by Image J software (NIH).

### Data analysis and functional annotations

LIMMA (PMID25605792), R software package (version 3.5.2) were used to screen differentially expressed proteins (|Log2Fc | > 0.5 and FDR ≤ 0.05).

The results were shown as the mean ± standard deviation. SPSS 23.0 statistical software was used for comparative analysis, the independent sample *t*-test was used for the analysis of differences between the two groups, and one-way analysis of variance (ANOVA) was used to evaluate the differences between multiple groups. The Pearson correlation coefficient was used to measure the correlation between two types of data. A *p-*value < 0.05 was considered to indicate statistical significance.

## Results

### Proteomics profiling analysis and validation of candidates by ELISA

**Figure 2:**
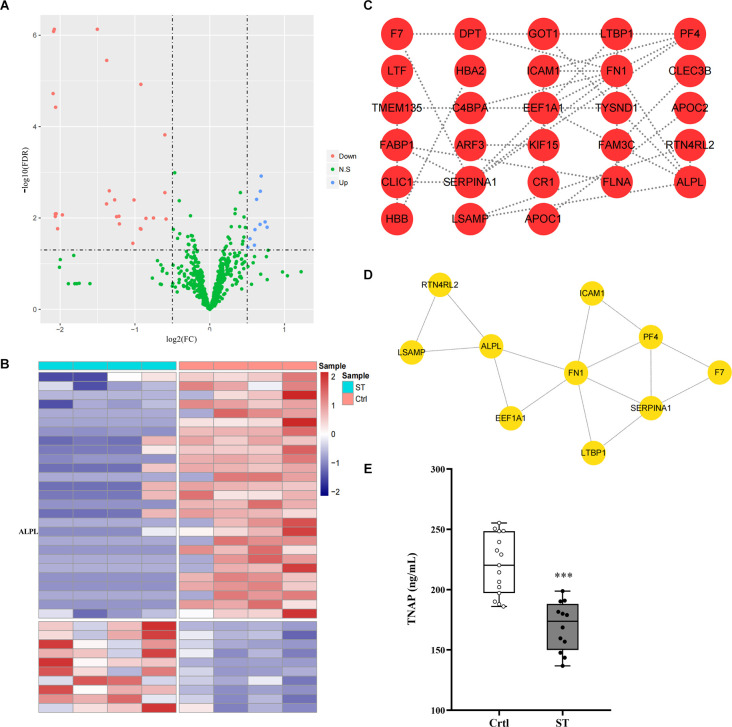
**(A,B)** Volcano map and heatmap showing 37 differentially expressed proteins between the ST group and the Ctrl group. The red and blue dots represented upregulated and downregulated proteins, respectively, and green represented proteins with no significant difference. The co-differential genes *RP1*, *ALPL*, and *FABP1* identified by expression profile chip detection and mass spectrometry detection were marked in panel **(B)**. **(C)** |Log2Fc| > 0.5 and FDR < 0.05 were used as the criteria for screening differentially expressed proteins. The PPI network diagram showed the differentially expressed proteins in the plasma of children in the ST group and the Ctrl group. Each dot in the network represented a protein, and the connection between the dots represented the relationship between them. The more connected lines there were, the more important the protein may be in the PPI network (minimum required interaction score > 0.4). **(D)** The top 10 genes in terms of degree in the PPI network screened by the MNC algorithm according to the test results of the plasma protein profiles of the children in the ST group and Ctrl group; the lines between the dots represented the relationship between them. **(E)** The content of TNAP in the plasma of children in the ST group and Ctrl group. Compared with that in the control group, the expression level of TNAP in the plasma of children in the spastic type CP group was significantly lower (*p* < 0.001, *n* = 12 for ST; *n* = 15 for Ctrl). ***p < 0.001 vs. Ctrl.

Four blood samples from each group from the same batch of the ST group and Ctrl group were used for protein profile detection, which identified 10 upregulated proteins and 27 downregulated proteins (Figures 2A,B; Supplementary Table S1). Then, the Search Tool for the Retrieval of Interacting Genes/Proteins (STRING) database was used to construct a protein-protein interaction (PPI) network of differential proteins (Figure 2C), and the top 10 important proteins were identified by the MNC algorithm, including *ALPL*/TNAP, *FN1*, *SERPINA1*, and *PF4* (Figure 2D).

To select candidates for validation, proteins were first chosen from our analysis described above. Meanwhile, blood samples from another group of 12 ST patients and 15 Ctrl children in the study cohort were collected which were not subjected to mass spectrometry analysis above. Compared with the Ctrl group, TNAP was significantly decreased in the ST group (*p* < 0.001, Figure 2E; Supplementary Table S2).

### GMFCS levels, blood count, and biochemical tests, MRI results of children with spastic CP

**Figure 3:**
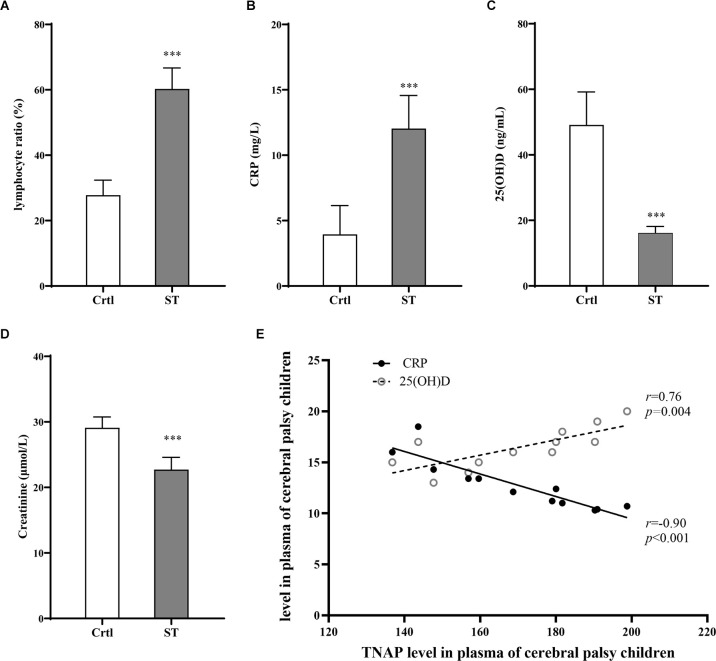
Results of blood tests for the Ctrl group (*n* = 20) and ST group (*n* = 18). **(A)** Compared with that of the Ctrl group, the percentage of lymphocytes in the ST group was significantly increased (*p* < 0.001). **(B)** The level of CRP in the ST group was significantly higher than that in the Ctrl group (*p* < 0.001). **(C)** Compared with those in the Ctrl group, the levels of 25 (OH)D in the ST group were significantly lower (*p* < 0.001). **(D)** The plasma Cr of the ST group was significantly lower than that in the Ctrl group (*p* < 0.001). **(E)** In the ST group, the level of TNAP was positively correlated with CRP (*p* < 0.001, *r* = −0.90) and 25 (OH)D (*p* = 0.004, *r* = 0.76). ***p < 0.001 vs. Ctrl.

The severity of motor impairment in the ST group (*n* = 18) was categorized by GMFCS grading. According to the GMFCS grading, there was no patient in grade I; four patients (22.2%) were in grade II, five (27.8%) were in grade III, six (33.3%) were in grade IV, and three (16.7%) were in grade V. Therefore, the participants at GMFCS Grade II and III which were classified as moderate motor dysfunction comprised 50% (*n* = 9) and those at Grade IV and V which were classified (*n* = 9) as severe motor dysfunction accounts for the other 50% (Table 2). Additionally, the percentage of peripheral blood lymphocytes and the contents of serum CRP, 25 (OH)D, and Cr were measured in both the ST group (18 participants) and the Ctrl group (20 participants). Compared with those in the Ctrl group, the percentage of lymphocytes and CRP levels in the ST group was significantly increased (*p* < 0.001, Figures 3A,B), while the 25 (OH)D and Cr levels were significantly reduced (*p* < 0.001, Figures 3C,D). Moreover, a direct association was also identified between the serum TNAP content and CRP, 25 (OH)D levels in the ST group (Figure 3E; Supplementary Table S3).

**Table 2:** Classification of the gross motor function in children with spastic CP.

Characteristics	ST (*n* = 18)	Control (*n* = 20)
**GMFCS level**	
*MMI*	9 (50%)	\
Level II	4 (22.2%)	\
Level III	5 (27.8%)	\
*SMI*	9 (50%)	\
Level IV	6 (33.3%)	\
Level V	3 (16.7%)	\

*MMI, moderate motor dysfunction; SMI, severe motor dysfunction*.

**Figure 4:**
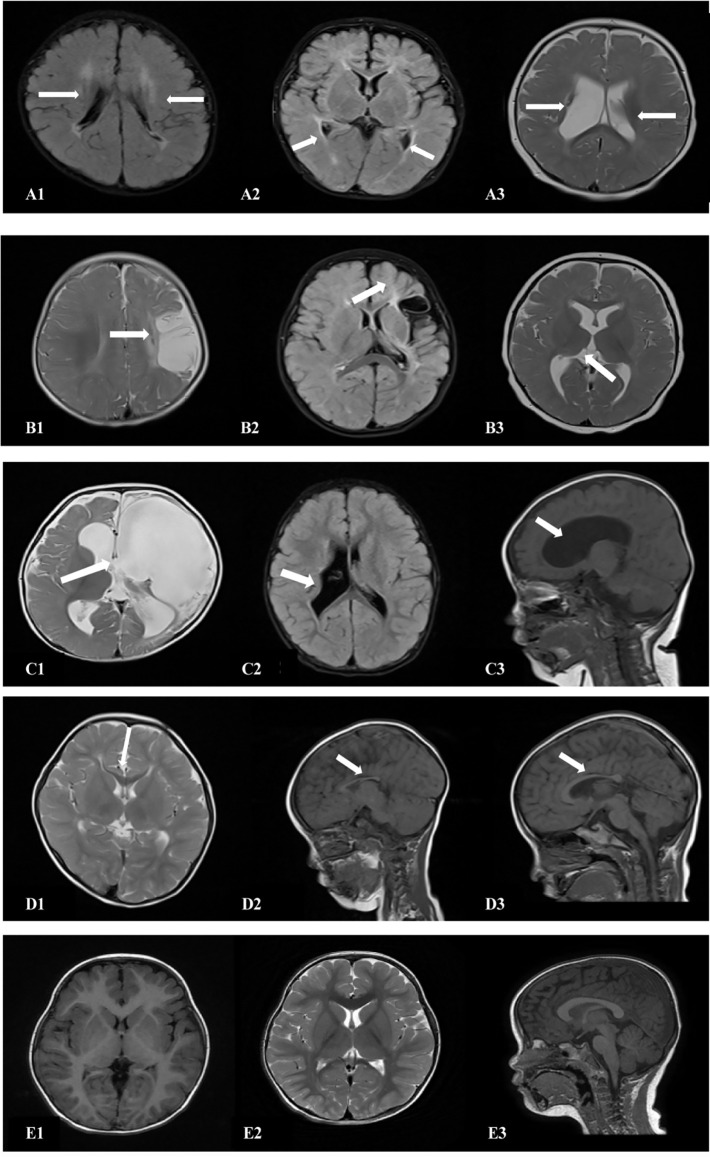
Cranial MRI findings of the 18 patients in the ST group. **(A1,2)** (transverse): Irregularly shaped bilateral ventricles with nearby, irregular, patchy T1 and T2 hypersignals. **(A3)** (transverse section): Enlarged bilateral ventricles with nearby, patchy T2 signals. **(B1)** (transverse section): Left frontotemporal parietal atrophy with subcortical softening foci. **(B2)** (transverse section): Reduced volume of the left basal ganglia. **(B3)** (transverse section): Decreased volume of the left thalamus. **(C1)** (transverse): Left partial cerebral perforation malformation with supratentorial hydrocephalus. **(C2)** (transverse section): Right ventricular para-body brain penetration malformation with surrounding gliosis. **(C3)** (sagittal section): Supratentorial hydrocephalus. **(D1)** (transverse section): Thinning of the corpus callosum. **(D2)** (sagittal): Noticeably thinner and shorter corpus callosum. **(D3)** (sagittal section): Significantly shorter corpus callosum. **(E1–3)** (TI/T2 transverse/sagittal): Normal brain MRI.

The cranial MRI findings of the 18 patients in the spastic group were as follows (Table 3): PVL was observed in 10 patients (55.6%), including three with ventriculomegaly and/or irregular shape of the lateral ventricles, four with abnormal signal foci in the ventricular white matter, and three with dysplasia of the corpus callosum. Cerebral atrophy was observed in two patients, supratentorial hydrocephalus in two patients, basal ganglia/thalamus lesions in two patients, porencephalia in one patient, and periventricular hemorrhagic infarction in one patient (Figure 4, Table 3).

**Table 3:** The appearance of brain MRI in children with spastic CP.

Cranial MRI findings	ST (*n* = 18)	Control (*n* = 20)
PVL	10 (55.6%)	\
ventriculomegaly	3	
abnormal signal foci	4	
dysplasia of corpus callosum	3	
Diffuse brain injury	6 (33.3%)	\
cerebral atrophy	2	
supratentorial hydrocephalus	2	
basal ganglia/thalamus lesions	2	
Focal lesion	2 (11.1%)	\
porencephalia	1	
periventricular hemorrhagic infarction	1	
Normal	\	20 (100%)

### Changes in TNAP and inflammation-related cytokines in the peripheral blood of rats with CP

After HIE modeling, infarction foci and liquefication or atrophy of the injured brain tissue (Supplementary Figures S1A, S1B) were observed in the Model group, and the amount of Nissl bodies was reduced relative to the Sham group (Supplementary Figure S2). From the day of modeling (P7), the weight of the pups in the Model group was significantly lower than that in the Sham group (*p* < 0.05; Figure 1B; Supplementary Table S4), and the righting reflex time of the Model group after modeling (P7–11) was significantly longer than that of the Sham group (*p* < 0.05; Figure 1C; Supplementary Table S5). On the 35th day post-HIE-modeling, the time for the Model group to pass the balance beam and the number of leg slips were significantly more than those of the Sham group (*p* < 0.001; Figures 1D,E; Supplementary Table S6). In sum, these results suggested that the HIE model rats mimicked the clinical and pathological changes in children with spastic CP and therefore modeling was successful.

**Figure 5:**
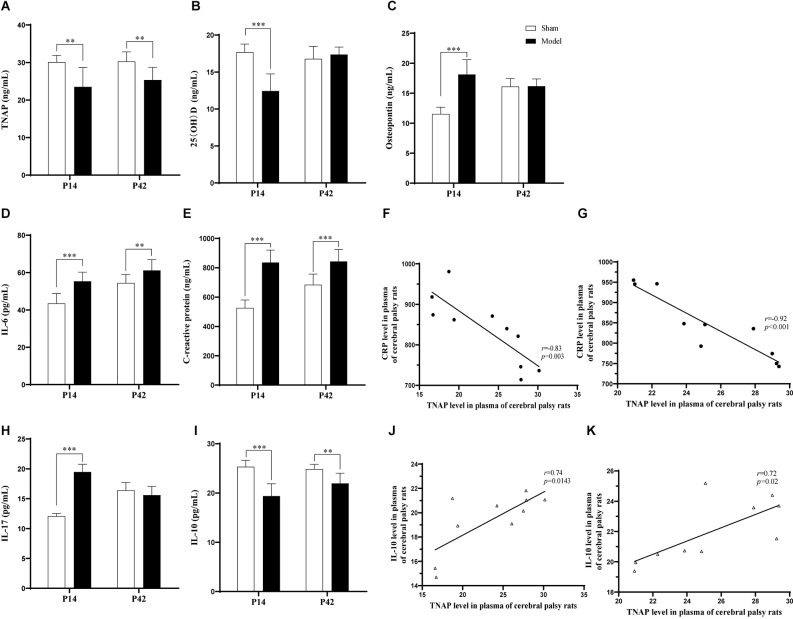
**(A,B,I)** Seven days after HIE modeling, compared with those in the Sham group, the levels of TNAP (*p* = 0.001, *n* = 10), 25 (OH)D (*p* < 0.001, *n* = 10), and IL-10 (*p* < 0.001, *n* = 10) were significantly reduced in the Model group. **(C–E,H)** The levels of OPN, IL-6, CRP, and IL-17 were significantly greater in the Model group than in the Sham group (*p* < 0.001, *n* = 10). **(F,J)** Seven days after HIE modeling, the plasma levels of TNAP were positively correlated with those of CRP (*p* = 0.003, *r* = −0.83, *n* = 10) and IL-10 (*p* = 0.0143, *r* = 0.74, *n* = 10). **(A,D,E)** Thirty-five days after HIE modeling, compared with the Sham group, the Model group had significantly lower levels of TNAP (*p* = 0.002, *n* = 10), IL-6 (*p* = 0.009, *n* = 10), and CRP (*p* < 0.001, *n* = 10), while the level of IL-10 was significantly decreased (*p* = 0.001, *n* = 10). **(G,K)** Thirty-five days after HIE-modeling, the plasma TNAP were correlated with CRP (*p* < 0.001, *r* = −0.92, *n* = 10) and IL-10 (*p* = 0.02, *r* = 0.72, *n* = 10). **p < 0.01 vs. Sham; ***p < 0.001 vs. Sham.

On the 7th day and 35th day post-HIE modeling, the levels of serum TNAP (*p* < 0.01, Figure 5A) and IL-10 (*p* < 0.001, *p* < 0.01, Figure 5I) in the Model group were significantly lower than those in the Sham group, while the levels of IL-6 (*p* < 0.001, *p* < 0.01, Figure 5D) and CRP (*p* < 0.001, Figure 5E) were significantly higher. Furthermore, the levels of OPN (*p* < 0.001, Figure 5C) and IL-17 (*p* < 0.001, Figure 5H) in pups in the Model group were significantly higher than those in pups in the Sham group while the serum 25 (OH)D content was significantly reduced on day 7 post-HIE (*p* < 0.001, Figure 5B). In addition, 7 days and 35 days after HIE modeling, the level of serum TNAP was correlated with those of serum CRP and IL-10 (Figures 5F,G,J,K; Supplementary Table S7).

### Changes in TNAP and inflammation-related cytokines in the brain tissue of rats with cerebral palsy

**Figure 6:**
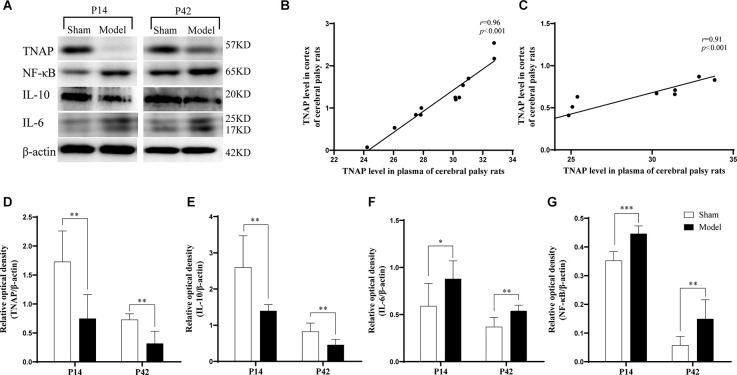
**(A)** Representative Western blot images of TNAP, IL-10, IL-6, and NF-κB in the injured cerebral cortex. **(D–G)** Compared with those in the Sham group, the cortex expression levels of TNAP and IL-10 were significantly reduced in the Model group (*p* < 0.01, *n* = 6). Compared with those in the Sham group, the levels of IL-6 (*p* < 0.05, *p* < 0.01, *n* = 6) and NF-κB (*p* < 0.001, *p* < 0.05, *n* = 6) were significantly increased in the Model group. **(B,C)** On day 7 (*p* < 0.001, *r* = 0.96, *n* = 12) and day 35 post-HIE-modeling (*p* < 0.001, *r* = 0.91, *n* = 12), there was a positive correlation in the levels of TNAP between the peripheral plasma and the brain. *Denotes statistical significance. *p < 0.05 vs. Sham. **p < 0.01 vs. Sham; ***p < 0.001 vs. Sham.

It was observed that the Model group displayed significantly lower expression levels of TNAP and IL-10 in the cortex on the injured side than the Sham group (*p* < 0.01; Figures 6A,D,E) while the IL-6 (*p* < 0.05, *p* < 0.01; Figure 6F) and NF-κB (*p* < 0.001, *p* < 0.01; Figure 6G) levels were significantly higher in Model group on day 7 and 35 post-HIE. Moreover, there was a strong positive correlation in TNAP levels between serum and brain tissue in HIE model rats both on the 7th and 35th days post HIE modeling (Figures 6B,C; Supplementary Table S8).

## Discussion

CP is not a disease in the traditional sense, but rather a clinical description of a series of signs and symptoms acquired during the antenatal, perinatal, or early postnatal period of life, due to a non-progressive brain injury, which may be shared by children or some adults (Kesar et al., 2012). Among several subtypes of CP, spastic CP is the most common one with the highest incidence. Whereas the molecular mechanism of the pathological changes in spastic CP is still unclear. In our study, proteomics profiling analysis was applied to analyze expressive levels of the genes involved in spastic CP. Ultimately, TNAP was screened out as the drastically down-regulated protein, which was in line with the findings in several spastic CP patients.

To confirm whether TNAP and CP were directly correlated, TNAP in the plasma and cerebral cortex of HIE model rats was evaluated. HIE model, the stable and widely used CP model demonstrates long-term ischemia and hypoxia which is consistent with the characteristics of spastic CP. Through Nissl staining and other methods, brain injury was observed in the model rats with CP, which was indicated by impaired motor function and muscle coordination in the righting reflex and balance beam tests. Together, these findings showed that the HIE model rats displayed clinical manifestations of CP successfully. Moreover, the results of our research were in line with the changes in the plasma of the children with CP, and there was a strong correlation between plasma TNAP and the cerebral TNAP in the injured cortex, which suggests that the changes in TNAP are related to CP occurrence.

The main biological function of TNAP is to hydrolyze the extracellular substrates inorganic pyrophosphate (PPi), pyridoxal-5-phosphate (PLP), and phosphor-ethanolamine (PEA; Mao et al., 2019). PLP is the main active form of vitamin B6 that exerts biological functions in the body (Zhang et al., 2021). TNAP is an ectoenzyme that is anchored to the outer cell membrane and to extracellular vesicles *via* its glycosyl-inositol-phosphate (GPI)-anchor. TNAP, not only has a high expression in the liver, kidney, and bone tissues but also plays an important role in the proliferation and differentiation of neurons during the development of the brain (Nwafor et al., 2021).

As to the role of TNAP in the pathological changes of spastic CP, a multitude of literature provided some leads. Graser et al. (2021) reported that TNAP contributes to the balance between proinflammatory ATP effects and the anti-inflammatory effects of its breakdown product adenosine, which has received attention. Different research teams have explored the mechanism of TNAP involvement in inflammation. As a start, Beck et al found that insufficient TNAP phosphatase activity leads to the accumulation of PPi and osteopathy, which can initiate the accumulation of calcium crystals in the joints and consequently initiate inflammatory processes (Beck et al., 2009). Subsequently, Akpinar (2018) showed that neuromuscular conditions such as CP may lead to vitamin D deficiency and under-nutrition in general. The major circulating metabolite of vitamin D, 25 (OH)D, is widely used as a biomarker of vitamin D status (Cashman et al., 2022); it can regulate the level of immune-inflammatory factors and plays an important role in the growth and development of the body. In addition, Huang et al. (2015) observed that 25 (OH)D can inhibit the production of inflammatory molecules in neuroglial cells by inhibiting the MAPK pathway and the production of downstream inflammatory molecules. In our study, the plasma levels of TNAP, 25 (OH)D, OPN, and Cr in children with spastic CP were significantly lower than those in the control group, which is consistent with the results of related studies.

Recently, some studies reported that OPN is a physiological substrate of TNAP and identified at least two preferred sites of dephosphorylation by TNAP. Yadav et al. (2014) found that low expression of TNAP led to elevated OPN, which can activate inflammatory factors such as IL-6 and IL-17 in bone cells, thus triggering inflammation. It has been reported (Albertsson et al., 2014) that OPN also promotes the secretion of proinflammatory cytokines and inhibits the production of anti-inflammatory factors such as IL-10. In this study, we also observed that a decrease of TNAP in children with spastic CP and in model rats triggered an increase in OPN expression, resulting in upregulation of IL-6, IL-17, and other proinflammatory factors in peripheral blood.

For the present, cranial MRI findings play an important role in evaluating the extension and severity of brain injury, detecting ischemia and/or diffuse axonal injury, and complementing the neurological evaluation (Pagnozzi et al., 2017). The European Cerebral Palsy Study reported (Ashwal et al., 2004) abnormal cranial MRI findings in 88.3% of patients, of which PVL was the most common, accounting for approximately 42.5%. The main manifestations of brain damage in spastic CP are PVL, and the main imaging findings are an irregular expansion of the lateral ventricles; paraventricular tissue softening; decreased white matter volume; and dysplasia of the corpus callosum. In our study, 10 (55.6%) of 18 children with spastic CP showed PVL on cranial MRI, and the lesion location was consistent with the literature mentioned above. The pathogenesis of PVL is multifaceted (Lawrence and Wynn, 2018; Cerisola et al., 2019; Magalhaes et al., 2019; Zhou et al., 2019), including maternal infection, cerebral ischemia, and vulnerability of brain white matter (Bax et al., 2006), among which the response of fetuses and newborns to inflammatory damage is the key. Hence, neuroinflammation, such as infiltration of leukocytes in the brain parenchyma along with activation of astrocytes and microglia can be observed in the brain of CP patients, which may cause further impairment of white matter. In recent years, Nwafor et al. speculated that TNAP’s regulatory phosphatase activity on a number of BBB endothelial proteins may play an important role in maintaining blood-brain barrier (BBB) integrity. Therefore, the decrease in TNAP expression levels in children with spastic CP may be one of the reasons for the pathological changes, such as BBB dysfunction and neuroinflammation (Nwafor et al., 2020).

Apart from neuroinflammation, there are also abnormal changes in the pro-inflammatory factors in the peripheral blood (Kaukola et al., 2004; Carlo et al., 2011; Patra et al., 2017). Some clinical studies suggested that anti-inflammatory therapy can improve the clinical manifestations of cerebral palsy (Chernykh et al., 2014; Allan, 2020). In this study, it was revealed that the levels of CRP and lymphocytes in the peripheral blood of children with spastic CP were significantly higher than those of the control group, indicating the occurrence of inflammatory reactions in the subject group. Similarly, the expression of CRP, IL-6, and IL-17 was significantly increased in the peripheral blood of model rats, while the level of the anti-inflammatory factor IL-10 was significantly decreased. We observed signs of brain damage, such as edema, atrophy, and liquefaction, in brain tissue slices of the injured cerebral hemisphere of model rats. Likewise, upregulation of IL-6 expression and downregulation of IL-10 expression were also detected in the cerebral cortex of model rats, confirming that inflammation is also involved in the brain injury process of spastic CP.

More importantly, downregulation of TNAP expression in the children with spastic CP along with low expression of TNAP and high expression of NF-κB in the cerebral cortex of HIE model rats were observed. In addition, the expression level of TNAP correlated well with CRP in both children with CP and HIE model rats. It was held by the previous study that TNAP is an inhibitor of NF-κB activity, and low expression of TNAP can induce NF-κB pathway activation (Hu et al., 2004). Meanwhile, it has been confirmed that the NF-κB pathway is a key transcriptional pathway in neuroinflammation caused by cerebral ischemia and hypoxia, which may promote the abnormal expression of inflammatory factors (Peng et al., 2022). Previous studies have suggested that cerebral ischemia and hypoxia can occur through the NF-κB/IL-6 pathway, resulting in rapid IL-6 release (Borsini et al., 2020). Moreover, IL-17 can activate downstream signaling pathways such as NF-κB, leading to the expression of proinflammatory chemokines and cytokines (Wang et al., 2021). Furthermore, McDonald et al. (2018) have reported that umbilical cord blood (UCB) cells therapy has well-documented neuroprotective effects on HIE model *via* anti-inflammatory effects.

Our research on the pathogenesis of TNAP in CP still requires to be further deepened and improved. For example, the effect of regulating the expression level of TNAP on the symptoms in CP model animals could be carried out in our follow-up research. Furthermore, a weakness of the present study is that TNAP is a relatively small protein with no known regulatory domains, and we could not obtain ALPL knockdown mice to use as animal models. Therefore, it is urgent to evaluate the inflammatory reactions and molecular mechanisms in *ALPL* knockdown mice.

In all, based on the analysis of the correlation between TNAP in peripheral blood and inflammatory factors and the high correlation between brain and peripheral blood TNAP, we suggest that the downregulation of TNAP expression in spastic CP might weaken the inhibition of NF-κB, leading to an abnormally high expression of NF-κB, the upregulation of the proinflammatory cytokines IL-6 and IL-17, and the downregulation of the anti-inflammatory factor IL-10, which contributes to the neuroinflammation in the progression of spastic CP. Our study was designed to pinpoint the chief molecule playing a specific and critical role in the neuroinflammation in spastic CP, which may provide a promising target for the diagnosis and treatment of spastic CP. To further our exploration, our future study will focus on the molecular mechanism of how TNAP regulates the pro-inflammatory factors related to CP neuroinflammation.

## Data Availability Statement

The data presented in the study are deposited in the ProteomeXchange Consortium repository, accession number PXD034911.

## Ethics Statement

The studies involving human participants were reviewed and approved by the ethics committee of Henan Province Children’s Hospital. Written informed consent to participate in this study was provided by the participants’ legal guardian/next of kin. The animal study was reviewed and approved by the National Institutes of Health Guide for the Care and Use of Laboratory Animals.

## Author Contributions

BL designed the study and revised the manuscript. X-KW performed experiments, analyzed the data and was a major contributor in writing the manuscript. CG acquired the clinical data. H-QZ, X-YK, and RQ performed the experiments and recorded the results. H-CZ, B-YC, and YG participated in data analysis. All authors contributed to the article and approved the submitted version.

## Funding

This work was supported by the National Natural Science Foundation of China (grant numbers: 81774444 and 82174522), Key special project of Traditional Chinese medicine research of Henan Province (20-21ZY1072), and international cooperation project of the Ministry of Science and Technology of China (G2021026025L).

## Conflict of Interest

The authors declare that the research was conducted in the absence of any commercial or financial relationships that could be construed as a potential conflict of interest.

## Publisher’s Note

All claims expressed in this article are solely those of the authors and do not necessarily represent those of their affiliated organizations, or those of the publisher, the editors and the reviewers. Any product that may be evaluated in this article, or claim that may be made by its manufacturer, is not guaranteed or endorsed by the publisher.

## Supplementary Material

SUPPLEMENTARY FIGURE 1 **(A)** When compared with sham group, the HIE rat’s cerebral infarct area was increased and the center of the infarction was uniformly pale after 48 h-HIE. **(B)** The brain tissue of the injured side of the cerebral palsy group was significantly liquefied and atrophy post 35 days-HIE.

SUPPLEMENTARY FIGURE 2 Representative images of the Nissl staining of pups. **(A–C)** The Nissl staining results in the Sham group (**B**: cortex; **C**: striatum). **(D–F)** The neuronal degeneration in the cortex and striatum after HIE in rat pups.

## Abbreviations

CP: cerebral palsy
CNS: central nervous system
ST: spastic typecerebral palsy
Ctrl: control
ALPL: Theliver/bone/kidneyalkaline phosphatase gene
TNAP: tissue-nonspecific alkaline phosphatase
HIE: hypoxic-ischemic encephalopathy
IL-6/17: interleukin-6/17
CRP: C-reactive protein
IL-10: interleukin-10
NF-κB: nuclear factor kappa-B
GMFCS-ER: The Gross Motor Function Classification System for cerebral palsy-Extended and Revised
MRI: Magnetic Resonance Imaging
T1W: T1-weighted
T2W: T2-weighted
PVL: periventricular leukomalacia
PWMI: periventricular white matter injury
LYM: percentage of lymphocytes
25 (OH)D: 25 hydroxyvitamin D
Cr: creatinine
P6 (P7,P11,P39,P42): postnatal day 6
TTC: triphenyl tetrazolium chloride
OPN: osteopontin
FC: fold change
FDR: false discovery rate
PPI: protein-protein interaction
MAPK: mitogen-activated protein kinase
PPi: inorganic pyrophosphate
PLP: pyridoxal-5-phosphate
PEA: phosphor-ethanolamine
GPI: glycosyl-inositol-phosphate
BBB: blood-brain barrier
MMI: moderate motor dysfunction
SMI: severe motor dysfunction.
