# Supplementary material for: TNAP—a potential cytokine in the cerebral inflammation in spastic cerebral palsy
Source: Front Mol Neurosci. 2022 Sep 14;15:926791. doi: 10.3389/fnmol.2022.926791 (PMC9515907; doi:10.3389/fnmol.2022.926791)
Supplement: Supplementary file 2 [file Table_2.docx]

**Supplement Table S2:The statistics of children's specific value of TNAP in peripheral blood**

|  | Ctrl (n=15) | ST (n=12) | P-value | t-value |
| --- | --- | --- | --- | --- |
| TNAP | 220.60±24.73 | 169.50±20.40^***^ | <0.001 | 5.76 |

***, *p*<0.001 vs. Ctrl

**Supplement Table S3: Statistics of clinical related indicators**

| Characteristics | Control (n=20) | ST (n=18) | P-value | t-value | Reference range |
| --- | --- | --- | --- | --- | --- |
| CRP, mg/L | 3.95±2.20 | 12.04±2.53^***^ | <0.001 | -10.53 | <10mg/L |
| Lymphocytes, % | 27.80±4.57 | 60.28±6.42^***^ | <0.001 | -18.11 | 20%-40% |
| 25(OH)D, nmol/L | 49.15±10.06 | 16.17±1.98^***^ | <0.001 | 13.66 | >50nmol/L |
| Creatinine, μmol/L | 29.10±1.65 | 22.72±1.87^***^ | <0.001 | 11.16 | 24.9-69.7μmol/L |

***, *p*<0.001 vs. Ctrl

**Supplement Table S4: Statistics of pups weight（g）**

| Postnatal days | Sham (n=10) | Model (n=10) | P-value | t-value |
| --- | --- | --- | --- | --- |
| P3 | 13.70±1.16 | 14.20±0.92 | 0.299 | -1.07 |
| P5 | 17.00±0.82 | 17.50±1.08 | 0.258 | -1.17 |
| P7 | 19.80±3.94 | 15.40±0.84^*^ | 0.003 | 3.45 |
| P9 | 23.60±4.30 | 18.00±1.33^*^ | 0.001 | 3.93 |
| P11 | 29.00±4.59 | 23.50±3.03^*^ | 0.005 | 3.16 |
| P13 | 33.60±4.81 | 29.60±3.17^*^ | 0.041 | 2.20 |
| P15 | 39.20±4.21 | 35.10±2.92^*^ | 0.021 | 2.53 |
| P17 | 46.80±3.58 | 42.20±3.97^*^ | 0.014 | 2.72 |
| P19 | 59.00±3.06 | 54.80±3.16^*^ | 0.007 | 3.02 |
| P21 | 72.50±2.32 | 66.00±3.40^*^ | <0.001 | 4.99 |
| P23 | 83.80±5.57 | 77.00±4.29^*^ | 0.007 | 3.06 |
| P25 | 98.30±4.08 | 88.80±6.00^*^ | 0.001 | 4.14 |
| P27 | 114.30±4.99 | 100.50±8.73^*^ | <0.001 | 4.34 |
| P29 | 127.80±8.22 | 112.20±8.89^*^ | 0.001 | 4.07 |
| P31 | 148.00±8.39 | 124.80±9.07^*^ | <0.001 | 5.94 |
| P33 | 167.00±7.06 | 135.50±10.74^*^ | <0.001 | 7.75 |
| P35 | 186.80±13.19 | 149.00±11.71^*^ | <0.001 | 6.78 |
| P37 | 199.70±12.45 | 161.40±11.06^*^ | <0.001 | 7.27 |
| P39 | 217.40±14.20 | 174.30±13.06^*^ | <0.001 | 7.06 |
| P41 | 235.70±13.46 | 183.90±16.72^*^ | <0.001 | 7.63 |

*, *p*<0.05 vs. Sham

**Supplement Table S5:The statistics of Righting reflex test （second）**

| Postnatal days | Sham (n=8) | Model (n=8) | P-value | t-value |
| --- | --- | --- | --- | --- |
| P6 | 2.19±1.01 | 2.38±0.64 | 0.666 | -0.44 |
| P7 | 1.46±0.39 | 4.87±3.57^*^ | 0.018 | -2.68 |
| P8 | 1.15±0.17 | 2.66±1.96^*^ | 0.048 | -2.17 |
| P9 | 1.36±0.33 | 2.12±0.67^*^ | 0.012 | -2.88 |
| P10 | 0.96±0.36 | 1.50±0.54^*^ | 0.033 | -2.36 |
| P11 | 0.70±0.18 | 1.20±0.27^**^ | 0.001 | -4.38 |

*, *p*<0.05 vs. Sham; **, *p*<0.01 vs. Sham

**Supplement Table S6: The statistics of Balance beam test**

| Postnatal days | Sham (n=8) | Model (n=8) | P-value | t-value |
| --- | --- | --- | --- | --- |
| Time to cross the balance beam, Second | 1.75±0.46 | 3.88±0.99^***^ | <0.001 | -5.50 |
| Number of slips of the hind limbs | 0.13±0.35 | 3.38±1.69^***^ | <0.001 | -5.34 |

***, *p*<0.001 vs. Sham

**Supplement Table S7: Statistics of related indexes in peripheral blood of pups**

|  | Postnatal days | Sham (n=10) | Model (n=10) | P-value | t-value |
| --- | --- | --- | --- | --- | --- |
| TNAP | P14 | 30.12±1.77 | 23.51±5.15^**^ | 0.001 | 3.84 |
|  | P42 | 30.26±2.60 | 25.35±3.36^**^ | 0.002 | 3.65 |
| 25(OH)D | P14 | 17.69±1.09 | 12.44±2.30^***^ | <0.001 | 6.54 |
|  | P42 | 16.78±1.68 | 17.37±1.00 | 0.357 | -0.95 |
| OPN | P14 | 11.53±1.12 | 18.13±2.45^**^ | <0.001 | -7.75 |
|  | P42 | 16.11±1.34 | 16.17±1.22 | 0.919 | -0.10 |
| CRP | P14 | 526.73±53.49 | 836.39±84.49^**^ | <0.001 | -9.79 |
|  | P42 | 684.91±72.23 | 843.60±81.50^**^ | <0.001 | 4.61 |
| IL-6 | P14 | 43.47±5.32 | 55.37±4.86^**^ | <0.001 | -5.23 |
|  | P42 | 54.39±4.57 | 61.17±5.80^*^ | 0.009 | -2.90 |
| IL-10 | P14 | 25.33±1.30 | 19.40±2.47^**^ | <0.001 | 6.73 |
|  | P42 | 24.87±0.95 | 21.96±2.06^*^ | 0.001 | -4.05 |
| IL-17 | P14 | 12.08±0.45 | 19.47±1.29^***^ | <0.001 | -17.15 |
|  | P42 | 16.40±1.31 | 15.60±1.45 | 0.211 | 1.30 |

*, *p*<0.05 vs. Sham; **, *p*<0.01 vs. Sham; ***, *p*<0.001 vs. Sham

**Supplement Table S8: Statistics of western blot gray value**

|  | Postnatal days | Sham (n=6) | Model (n=6) | P-value | t-value |
| --- | --- | --- | --- | --- | --- |
| TNAP/β-actin | P14 | 1.73±0.53 | 0.75±0.41^*^ | 0.005 | 3.60 |
|  | P42 | 0.73±0.10 | 0.32±0.21^*^ | 0.001 | 4.40 |
| IL-10/β-actin | P14 | 2.60±0.87 | 1.40±0.17^*^ | 0.008 | 3.31 |
|  | P42 | 0.83±0.23 | 0.46±0.15^*^ | 0.008 | 3.27 |
| IL-6/β-actin | P14 | 0.59±0.24 | 0.88±0.19^*^ | 0.047 | -2.26 |
|  | P42 | 0.37±0.10 | 0.54±0.06^*^ | 0.003 | -3.80 |
| NF-κB/β-actin | P14 | 0.35±0.03 | 0.45±0.03^**^ | <0.001 | -5.56 |
|  | P42 | 0.06±0.03 | 0.15±0.07^*^ | 0.012 | -3.07 |

*, *p*<0.05 vs. Sham; **, *p*<0.01 vs. Sham; ***, *p*<0.001 vs. Sham
