# Supplementary material for: TNAP—a potential cytokine in the cerebral inflammation in spastic cerebral palsy
Source: Front Mol Neurosci. 2022 Sep 14;15:926791. doi: 10.3389/fnmol.2022.926791 (PMC9515907; doi:10.3389/fnmol.2022.926791)
Supplement: Supplementary file 4 [file Presentation_1.PPTX]

## Slide 1
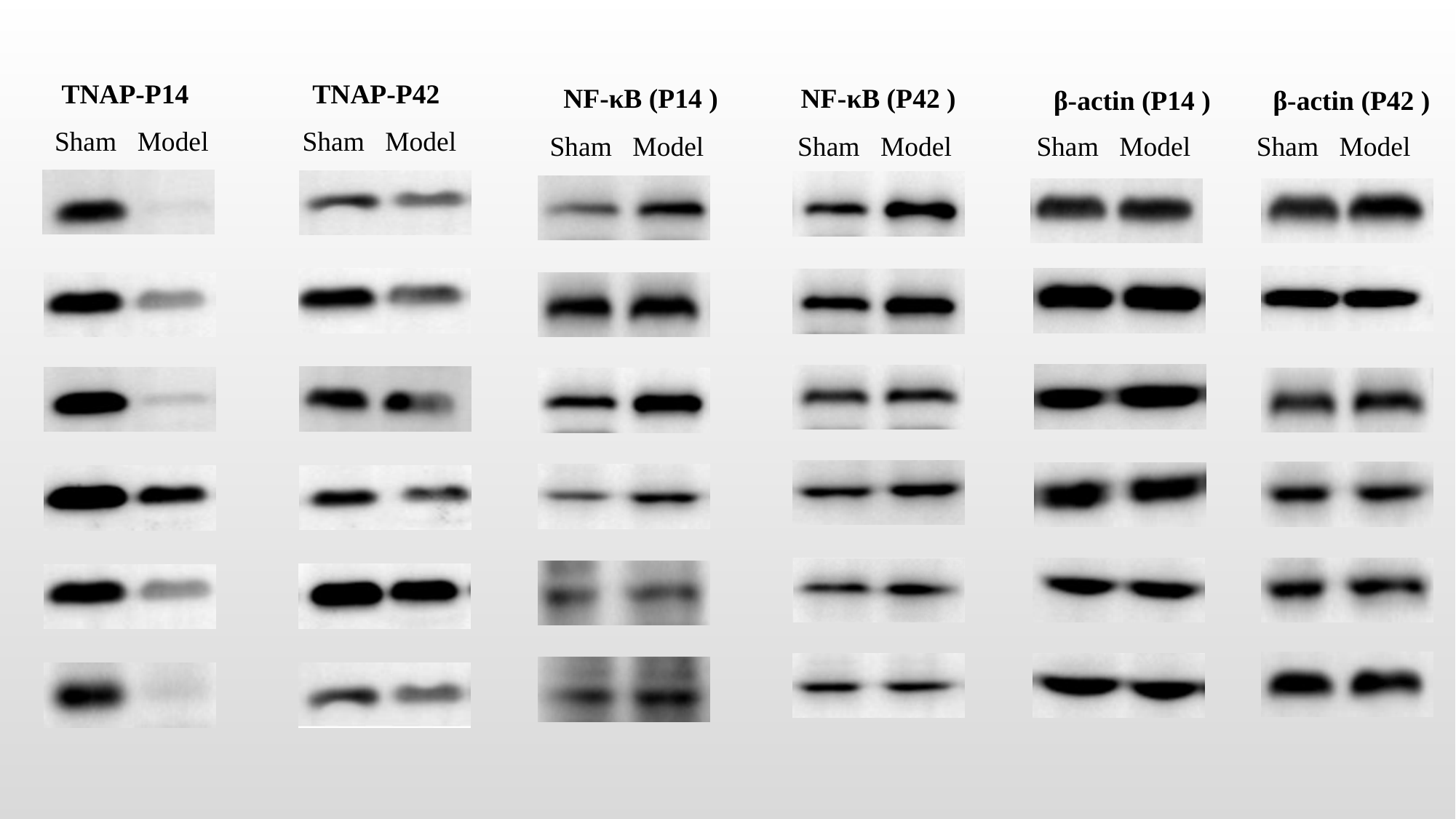

TNAP-P14 TNAP-P42
Sham Model
Sham Model
 NF-κB (P14 ) NF-κB (P42 )
Sham Model
Sham Model
 β-actin (P14 ) β-actin (P42 )
Sham Model
Sham Model

## Slide 2
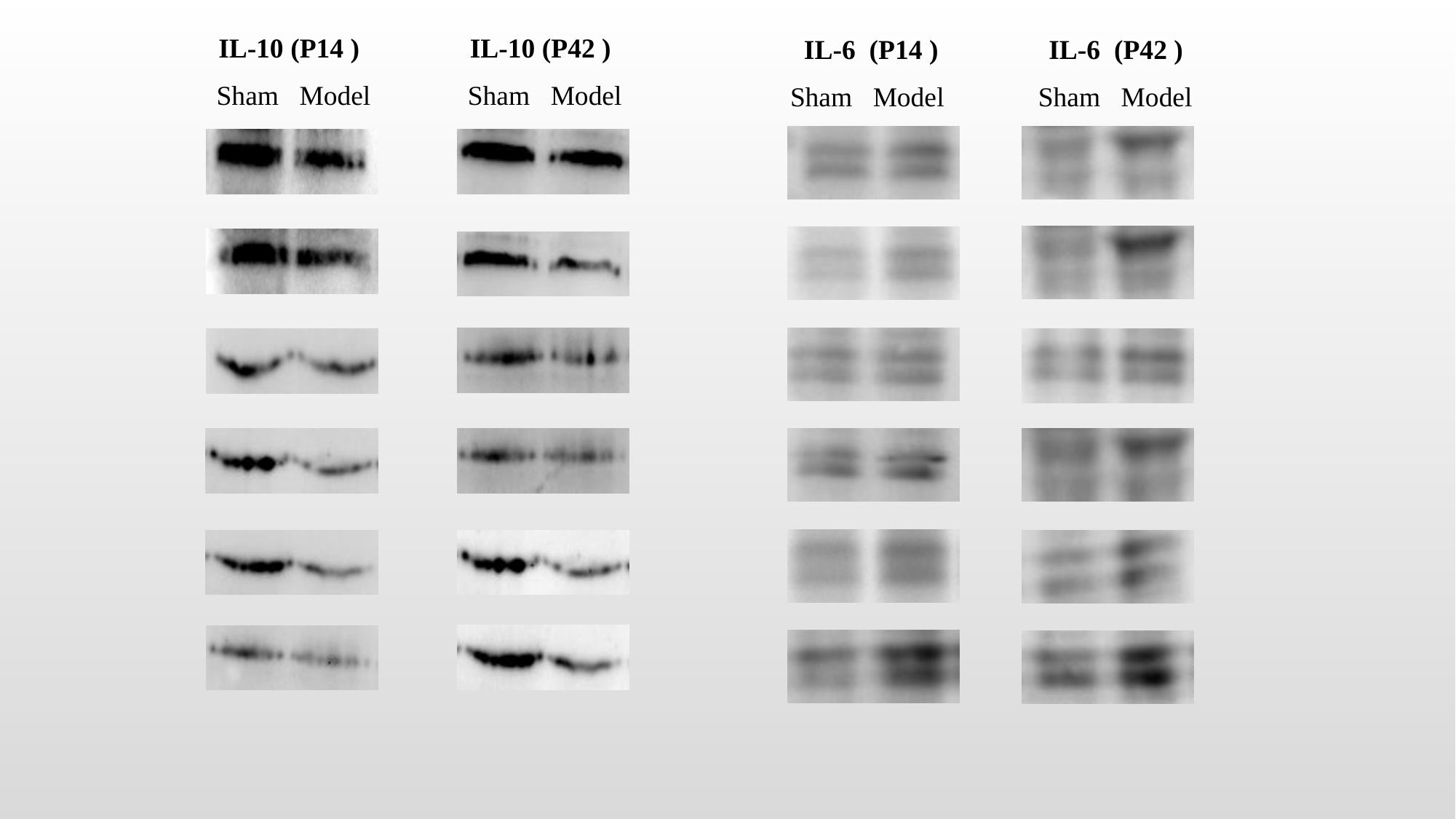

IL-10 (P14 ) IL-10 (P42 )
Sham Model
Sham Model
 IL-6 (P14 ) IL-6 (P42 )
Sham Model
Sham Model
