# Supplementary figures and images for: TNAP—a potential cytokine in the cerebral inflammation in spastic cerebral palsy
Source: Front Mol Neurosci. 2022 Sep 14;15:926791. doi: 10.3389/fnmol.2022.926791 (PMC9515907; doi:10.3389/fnmol.2022.926791)

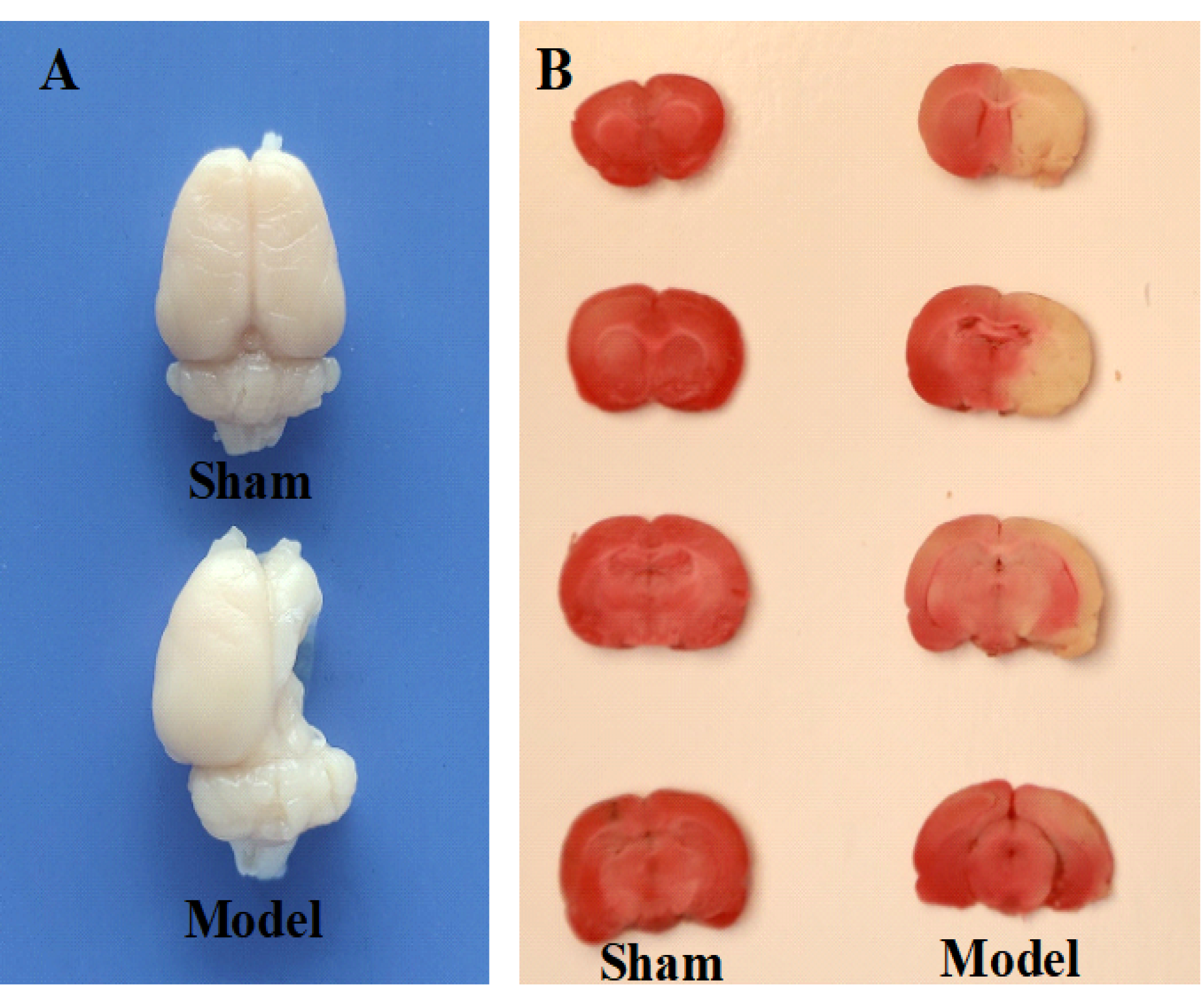

Supplement: Supplementary file 6 [file Image_1.TIF]

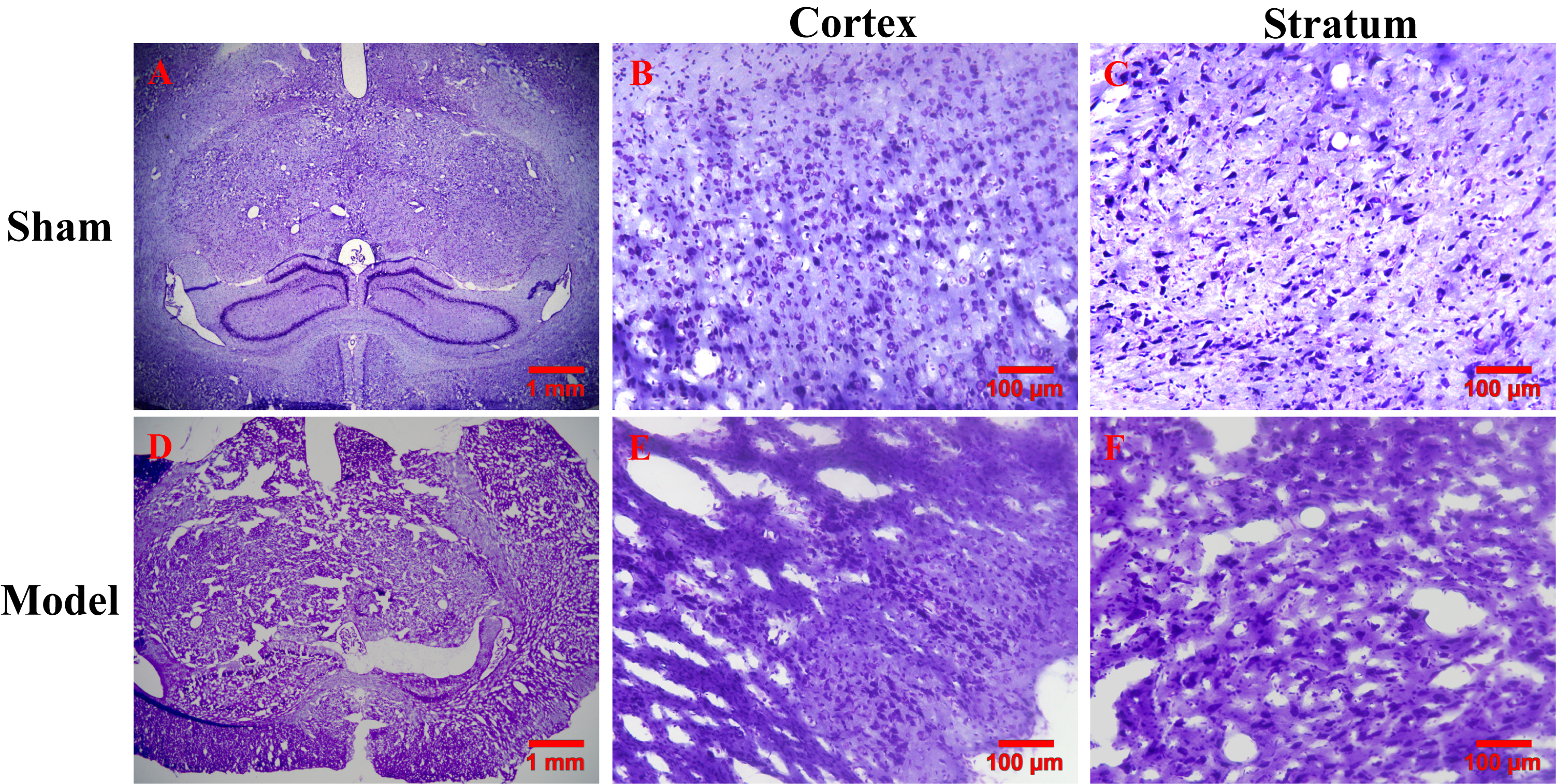

Supplement: Supplementary file 7 [file Image_2.TIF]
